# Role of Preoperative Oral Rehydration Solution on Myocardial Ischaemia During Orthopaedic Surgery under Spinal Anaesthesia: A Prospective Randomised Study

**DOI:** 10.4274/TJAR.2023.231206

**Published:** 2023-10-24

**Authors:** Hithish MJ, Gaurav Jain, Priyanka Gupta, Roop Bhushan Kalia, Praveen Talawar

**Affiliations:** 1 Department of Anaesthesiology and Critical Care, All India Institute of Medical Sciences, Rishikesh, Uttarakhand, India; 2 Department of Orthopaedics, All India Institute of Medical Sciences, Rishikesh, Uttarakhand, India

**Keywords:** Fasting, geriatrics, myocardial ischaemia, ORS, orthopaedics, spinal anaesthesia

## Abstract

**Objective:**

Preoperative oral rehydration solution (ORS) supplementation offers wide postoperative benefits, but its role in reducing post-spinal myocardial ischaemia is uncertain. We evaluated this aspect in elective lower limb orthopaedic surgeries and compared it to conventional preoperative fasting.

**Methods:**

Prospectively, we randomised 126 patients aged >60 years into two groups: (A) received reconstituted ORS (1000 mL) during the overnight preoperative fasting, continued up to 2 hrs prior to spinal anaesthesia (SA) induction; (B) kept on conventional overnight preoperative fasting. This study evaluated electrocardiographic ischaemic changes at 2, 5, 10, 15, and 30 minutes after SA induction.

**Results:**

In total, 27 patients (group A: 7; group B: 20) developed transient electrocardiographic ischaemic changes. On intergroup comparison, group B had a significantly higher incidence at all time points, with highest statistical levels at 5- and 10-minutes ((*P* < 0.001). The receiver operating characteristic curve at a threshold fasting duration (fluids) of >3 hours, had an area-under-curve of 0.74 to predict such changes within 30 minutes of SA induction (sensitivity 96.30%, specificity 55.56%, accuracy 64.29%, odds ratio 32.50, relative risk 20.80, (*P* < 0.001). Post-spinal hemodynamic changes were higher in group B than in A; hypotension and tachycardia were statistically significant ((*P*=0.020). The pleth variability index was significantly higher ((*P* < 0.001), while perfusion index was significantly lower (*P* < 0.001) in group B at all time points.

**Conclusion:**

Preoperative ORS supplementation significantly reduced post-spinal transient ischaemic electrocardiographic changes in elderly patients than conventional overnight fasting.

**Main Points**

• Preoperative oral rehydration solution supplementation significantly reduced the post-spinal ischemic electrocardiographic (ECG) changes compared to conventional overnight fasting in elderly patients undergoing elective lower limb orthopaedic surgery under spinal anaesthesia (SA).

• It was coherent with unstable haemodynamics and abnormal perfusion parameters at the same intraoperative time points.

• No patient complaint of notable anginal symptoms and the underlying ischaemic ECG changes also reverted within an hour of SA induction.

• The threshold fasting duration of >3 hours for oral fluids had an area-under-curve of 0.74 to predict ischaemic ECG changes within 30 minutes of SA induction.

## Introduction

Preoperative fasting is a prerequisite for most surgical procedures. It aims to minimise the gastric residual volume (GRV) to avoid aspiration of gastric contents, a potential cause of airway-related perioperative mortality.^1^ The underlying mechanism includes reduced sympathetic tone under spinal anaesthesia (SA). However, the flip-side is increased patient hunger, anxiety, insulin resistance, hyperglycaemia, unstable intraoperative haemodynamics, cardiac ischaemia, altered thermoregulation, and decreased cardiac preload, especially in elderly patients.^2^ Literature, however, shows no difference in GRV between fasted patients and those receiving preoperative fluids.^3^ Guidelines also recommend fluids up to 2 hrs prior to surgery.^1^ However, in clinical practice, preoperative fasting usually lasts for ≥10 hours due to early patient shifting to the preoperative area during the morning hours, insufficient staffing to look for the preoperative fasting hours, and legal fear and apprehension among surgeons for case cancellation due to inadequate fasting.^3,4^

Though preoperative clear fluids may alleviate patient dehydration, complications related to electrolyte and sugar imbalance are still unaccounted. Formulations including carbohydrate drinks and oral rehydration solution (ORS) help to preserve perioperative muscle mass, reduce anxiety, hunger, and postoperative pain, maintain nitrogen balance, and allow early patient discharge.^5,6^ However, literature showing its effect on myocardial ischaemia after SA induction is relatively sparse. We hypothesised that preoperative ORS supplementation might reduce the risk of post-spinal myocardial ischaemia. We explored the effect of preoperative ORS supplementation on myocardial ischaemia at different time points during the first 30 minutes after SA induction in patients undergoing elective lower limb orthopaedic surgery, compared to traditional overnight preoperative fasting. Other parameters, including pleth variability index (PVI), perfusion index (PI) and intraoperative complications were also compared at same time points.

## Methods

After All India Institute of Medical Sciences, Rishikesh, Institutional Ethics Committee approval (approval no: 331/IEC/PGM/2020, date: 20.06.2020) and written informed consent, 126 patients aged >60 years, of either sex, American Society of Anesthesiology grade I-III, undergoing elective lower limb orthopaedic surgery under SA admitted between November 2020-April 22, were included in this prospective, outcome-assessor blinded, interventional trial. We followed all ethical principles for medical research involving human subjects as per Helsinki Declaration 2013. We excluded those with known cardiac/renal disease, diabetes mellitus, body mass index >35 kg m^-2^, requiring emergency surgery, unable to give consent, any contraindication to SA or evidence of ischaemic changes/electrocardiographic (ECG) signs mimicking myocardial ischaemia in 12-lead ECG/echocardiogram during pre-anaesthetic check-up (PAC) prior to surgery.

The included patients were randomised into two groups, “A or B” (1:1 allocation ratio), using a computer-generated random sequence, and concealed via serially numbered sealed-opaque-envelope technique. An investigator performing patient randomization, the investigator assigned to prepare ORS solution/perform preoperative work-up/gastric ultrasound, the supervising anaesthetist, and the outcome assessor remained blinded to other aspects. All patients received similar perioperative care as per standard protocols.

Group A: received 1 litre of reconstituted ORS [Oral rehydration salts sachet (Sodium chloride 2.6 g, Potassium Chloride 1.5 g, Sodium Citrate 2.9 g, Dextrose 13.5 g), Bureau of Pharma PSUs of India, mixed in 1 litre of water], administered overnight after dinner, prior to the planned day of surgery, and up to 2 hrs prior to SA induction next morning.

Group B: kept on traditional overnight fasting (solids and liquids) starting after dinner before the planned day of surgery and continued to SA induction the next morning.

In the preoperative room, IV access was established, and patients received premedication with Ranitidine 50 mg IV and Metoclopramide 10 mg IV. The GRV was measured 1/2 an hour before the scheduled surgery using a curvilinear ultrasound probe (GE parallel Design Inc. Phoenix, US, 4C-RS, Frequency range 2-5 MHz), first under supine and then right lateral position. The GRV was calculated as 27.0 + [14.6 x cross-sectional area (CSA) (cm^2^)] - [1.28 x age (years)]. The antral CSA was measured using two perpendicular diameters and the area ellipse formula [CSA = (Anteroposterior diameter x craniocaudal diameter x 22/7)/4].^7^ A GRV threshold ≤1.5 mL kg^-1^ was considered adequate to proceed with surgery; if observed GRV was >1.5 mL kg^-1^, we reassessed such patients on completion of another surgery posted for the day.

Patients who met GRV criteria were shifted to the operation theatre. A multipara monitor, digital 12-lead ECG machine, and Masimo Radical 7 device (Rev E, Masimo Corp., Japan) were attached, IV fluid [Ringer lactate (RL) 15 mL kg^-1^ co-loaded over 30 minutes] started, and baseline parameter recorded. The lower limb electrodes of 12-lead ECG were placed on anterior axillary line, halfway between costal margin and iliac crest, bilaterally. With patients under a seated position, SA was induced at L3-L4/L4-L5 vertebral level with a 25G Quincke spinal needle using hyperbaric Bupivacaine 0.5% (2.5 mL) with Fentanyl 25 µg (0.5 mL) under appropriate aseptic precautions. The time point just after drug injection in a spinal block was taken as “0 min”. Patients were then turned supine. The supervising anaesthetist looked after the intraoperative IV fluid need. After the RL co-load, if PVI was >13% over 5 minutes, a 250 mL IV RL bolus was administrated over 10 minutes and repeated until PVI reached ≤13%. If PVI was ≤13%, RL infusion was continued at 2 mL kg^-1^ hr^-1^ IV. Surgery was allowed to proceed after achieving a T10 sensory level to loss of cold sensation to alcohol-soaked gauge. For any hypotensive episode (MAP ≤90% of baseline), patients were treated with IV fluid augmentation (250 mL IV RL bolus) and Phenylephrine 50-100 µg IV bolus, as required. Bradycardia (heart rate ≤50 beats min^-1^) was treated with. Atropine 0.5 mg IV. Hypoxemia (SPO_2_ <94% or respiratory discomfort) was treated with free flow oxygen via facemask. Any hypertensive episode (MAP ≥10% from baseline) was treated with Labetalol 5 mg IV bolus dose.

The primary outcome included ischaemic changes in 12-lead ECG [new-onset T-wave inversion or ST-segment elevation/depression (≥0.1 mm mV)] in any cardiac lead measured at 2, 5, 10, 15 and 30 minutes after SA induction, and compared to baseline PAC ECG. The secondary outcome included PVI/PI measured at the same time points and post-spinal complications (bradycardia, tachycardia, hypotension, hypertension, hypoxia) within first 30 minutes of SA induction. Other recorded variables included preoperative fasting duration, level of sensory block at 20 min after SA induction, intraoperative IV fluid infused, surgery duration, and intraoperative blood loss.

### Statistical Analysis

The sample size was calculated using Open-Epi Collection of Epidemiologic Calculator 3.01 (Andrew G. Dean, Kevin M. Sullivan, Atlanta, GA, US). The primary sample size calculation was based on expected 12-lead ECG ischaemic changes (within 30 minutes of SA induction) in 20% of patients receiving preoperative fasting and 2% in those receiving ORS during the fasting phase prior to elective lower limb orthopaedic surgery (pilot observations). Using “Fleiss with CC” model with 95% confidence level and 80% power, the sample size was calculated as 57 per group (114 patients). Considering a 10% dropout, we required 63 patients per group. For statistical analysis, we used a Statistical Package for Social Sciences software 23.0 (SPSS, IBM Corp, Armonk, NY, US). We assessed the normality of data by Kolmogorov-Smirnov test and summarised the results as mean (standard deviation), median (range), or frequency (%). Inter-group comparison for continuous variable was performed by Mann-Whitney U test or Unpaired t-test, as per Gaussian distribution. The categorical variable was analysed by chi-square test or Fisher’s exact test. The value of preoperative fasting duration (fluids) in predicting myocardial ischemia (within 30 minutes after SA induction) was analysed by the receiver operating characteristic (ROC) curve. A *P* value < 0.05 was considered significant.

## Results

**Figure 1:**
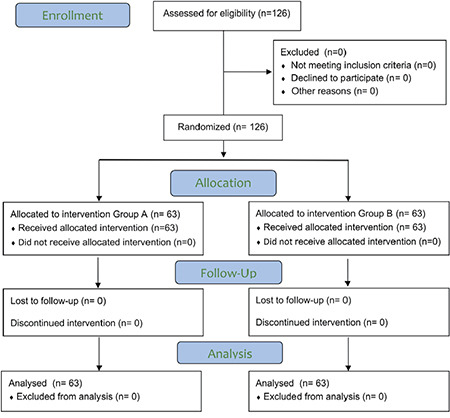
CONSORT flow diagram of patients studied.

We included 126 eligible patients over 18 months, with no dropouts (Figure 1). The baseline patient profile was comparable among the groups, with a majority within 60-70-year age group, predominantly males, ASA grade I-II, of average build-up, and significant comorbidity being hypertension. The major indication for surgery included total knee replacement and fracture reduction. The duration of fasting for solids was comparable, while that for fluids was statistically significant among groups. The GRV was well below the threshold criteria in all included patients and comparable among the groups (Table 1).

**Figure 2:**
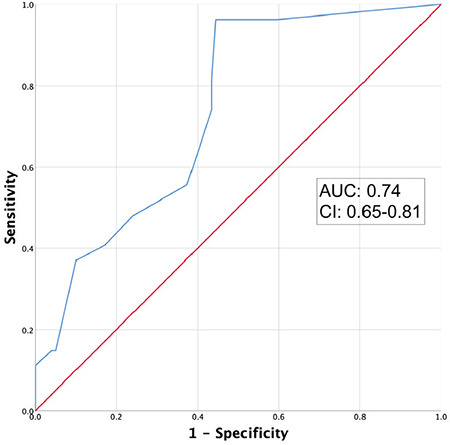
Receiver operating characteristic curve showing the utility of fasting duration (fluids) as predictor of myocardial electrocardiographic ischaemic changes within 30 minutes after spinal anaesthesia induction. AUC, area under curve; CI, confidence interval.

In total, 20 patients in group B and 7 in group A developed myocardial ECG ischaemic changes within 30 minutes after SA induction. Taking different time points individually, 16 patients in Group B had such changes at 2 and 5 minutes, 20 at 10 minutes, 15 at 15 minutes, and 10 at 30 minutes after SA induction, respectively. In group A, 5 patients had such changes at 2 minutes, 2 at 5 minutes, 4 at 10 minutes, and 3 at 15 and 30 minutes after SA induction, respectively. On intergroup comparison, statistical difference was observed at all studied time points, with highest significance achieved at 10 minutes after SA induction (*P *< 0.001) (Table 2). The ROC curve had an area-under-curve of 0.74 (confidence interval=0.65-0.81,* P *< 0.001) at a threshold fasting duration (fluids) of >3 hours to predict myocardial ECG ischaemic changes at any timepoint within 30 minutes of SA induction (sensitivity 96.30%, specificity 55.56%, accuracy 64.29%) (Figure 2). The relative risk (RR) for such changes at the above threshold was 20.80 (2.91-148.58, *P*=0.002).

A median sensory level of “T8” was noted 20 minutes after SA induction, which was comparable among the groups (Table 1). The PVI was significantly higher in group B as compared to A at all time points (*P *< 0.001); On the contrary, PI was significantly higher in group A at corresponding time points (*P *< 0.001) (Table 2). The major hemodynamic changes within 30 minutes after SA induction included bradycardia in 7 versus 9 patients, tachycardia in 14 versus 26, hypotension in 13 versus 25, and hypoxia in 1 versus 4 patients in Group A and B, respectively. On intergroup comparison, only hypotension (*P*=0.020) and tachycardia (*P*=0.022) attained statistical significance (Table 2).

## Discussion

We observed that patients receiving ORS solution during the preoperative fasting phase (for solids) had a significantly lower incidence of transient myocardial ECG ischaemic changes during the first 30 minutes after SA induction compared to those receiving overnight preoperative fasting (both for solids and fluids) for elective lower limb orthopaedic surgery. Its coherence to unstable haemodynamics and abnormal perfusion parameters during the same studied time points further supports the above findings.

During the preoperative fasting phase, body fluids depreciate primarily via insensible fluid loss and urine production, leading to relative hypovolemia. After SA induction, this aggravates post-spinal hypotension, which causes a sudden decrease in coronary artery perfusion pressure and may trigger transient myocardial ischaemia, even if we co-load the patients with IV fluids.^4^ This deviation reflects as transient T-wave inversion or ST segment changes in the ECG. An aggravated sympathetic tone resulting from preoperative anxiety and stress may further supplement such changes and consequent complications.^8^ We observed that fasting duration for fluids >3 hours significantly predicted myocardial ischaemic changes in the ECG, including all the studied time points (area under curve=0.74), with high odds ratio (32.5) and hazard ratio (20.8) to predict such changes. The observed changes were significantly high in overnight fasted patients (31.7%) having a fasting duration of 10-14 hours (solids or liquids). Yeniay et al.^4^ also observed that prolonged fasting (10-18 hours) was significantly associated with intraoperative cardiac ischaemic changes (20.8%) and decreased mean arterial pressure and heart rate in a similar subset of patients. A higher incidence in our study could be related to a difference in patient demographics, ethnicity, comorbidities, anxiety level and other unaccounted factors. However, both groups were comparable regarding such differences and preoperative preparation.

The degree of dysglycemia is another independent risk factor contributing to ischaemic cardiac events during the perioperative period, especially in diabetic patients.^9^ Although preoperative IV glucose supplementation is considered efficacious, a high dosage is required for sufficient insulin release that could minimise catabolic effects.^10^ Moreover, IV preload/co-load over short periods, especially in elderly patients, may cause fluid overload with overt complications.^11^ Oral replacement is a good alternative if rehydration solution has sufficient carbohydrate concentration (>12.5%) with lower osmolality (265 osmole L) to allow rapid insulin release and gastric emptying.^10^ We chose preoperative ORS considering this assumption. Due to its comprehensive postoperative benefits, preoperative carbohydrate drink is now integral to enhanced recovery after surgery protocol for fast-track surgeries.^12^ We observed significantly lower post-spinal ischaemic ECG changes in the ORS group. None of the patients in either group complained of notable anginal symptoms, and ECG changes also reverted within an hour of SA induction. Nevertheless, such changes may have particular significance in high-risk groups like diabetes mellitus and coronary artery disease, where cardiac ischaemia may progress to infarction. We noted such changes for only 30 minutes of SA induction to obviate the effects of surgical factors.

We observed a significantly higher incidence of hypotension and tachycardia in the fasted group compared to those receiving preoperative ORS; a significant difference in intraoperative IV fluid infusion further support these findings. We observed most of such episodes within 10 minutes after SA induction, coherent with ischaemic ECG changes in both groups at the “10-minute” time point. Other complications like bradycardia, and hypoxia, though higher in fasted patients, were comparable among groups. Itoh et al.^13^ also observed that preoperative ORS significantly minimises a decrease in systolic blood pressure and hypotensive episodes after SA induction.To investigate the effect of dehydration on systemic tissue perfusion, we also measured PI and PVI values at the same time points. The PVI was significantly higher, and PI was significantly lower at all studied time points in fasted patients compared to the ORS group. Emektar et al.^14^ also observed that moderate to severe dehydration leads to lower PI and higher PVI values in acute gastroenteritis patients. Tsutsui et al.^15^ observed that preoperative ORS increases the circulating blood volume and keeps a higher cardiac index during induction of general anaesthesia, reducing any intraoperative hypotensive episodes. Thus, preoperative dehydration reduces peripheral tissue perfusion, which may also contribute to cardiac ischaemia.

### Study Limitations

Our study has a few limitations. We planned to include total knee replacement patients only, but due to the ongoing coronavirus disease-2019 pandemic, we incorporated other elective lower limb orthopaedic surgeries to achieve the desired study sample in a restricted timeframe. However, in view of equivalent intraoperative parameters, this study generates sufficient data highlighting the utility of preoperative ORS in reducing post-spinal myocardial ischaemia. Secondly, preoperative anxiety could also account for such ECG changes. However, routine use of anxiolytics and preoperative counselling in all included patients minimised any intergroup differences. Finally, we could not account for various outcome parameters like perioperative insulin resistance and immune status due to logistic issues. A future trial on a larger sample size may delineate such aspects.

## Conclusion

In conclusion, preoperative ORS supplementation significantly reduced the transient post-spinal ischaemic ECG changes in elderly patients compared to conventional overnight fasting, during lower limb orthopaedic surgery. A dedicated perioperative team to care for issues like patient fasting status may add a difference to perioperative outcome, especially in high-risk groups with minimal vital reserve.

## Figures and Tables

**Table 1:**
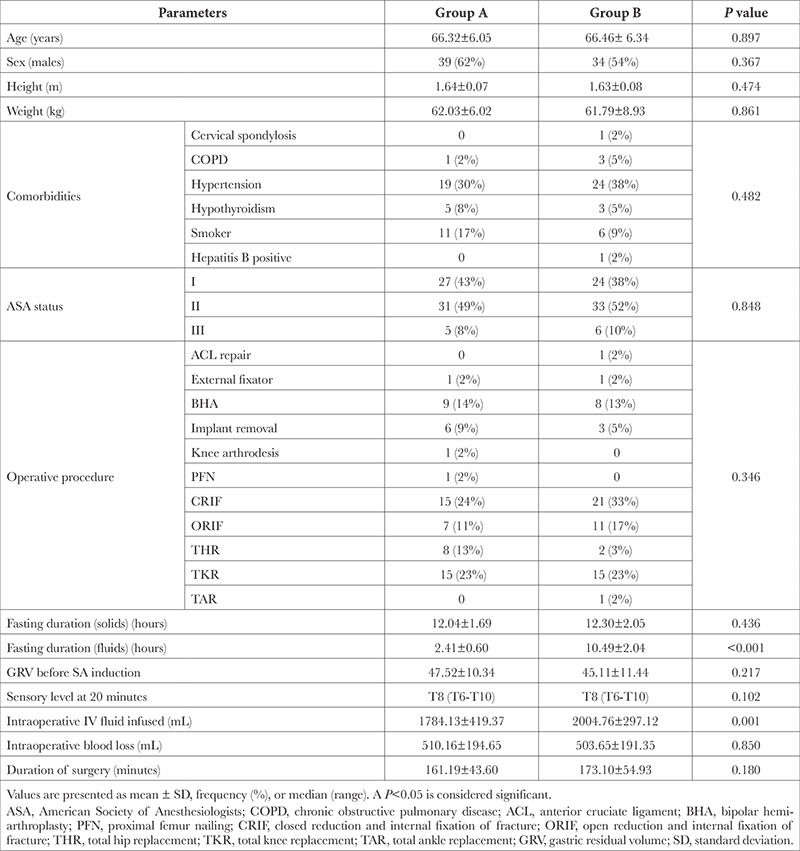
The Baseline and Outcome Parameters of the Included Patients (n = 126)

**Table 2:**
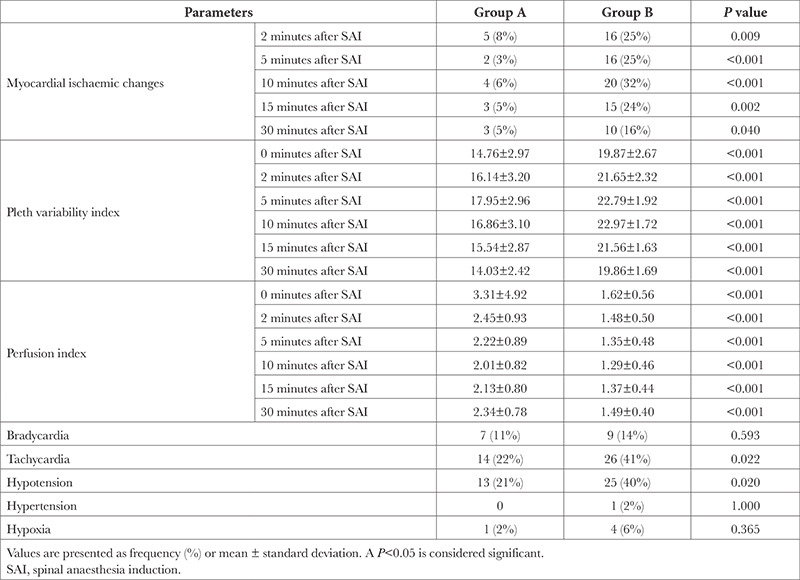
Myocardial Electrocardiographic Ischaemic Changes, Perfusion Parameters and Cardiorespiratory Complications Within 30 Minutes of Spinal Anaesthesia Induction Among Included Patients (n = 126)
